# Metabolomics Analysis of the Lipid-Regulating Effect of* Allium hookeri* in a Hamster Model of High-Fat Diet-Induced Hyperlipidemia by UPLC/ESI-Q-TOF Mass Spectrometry

**DOI:** 10.1155/2018/5659174

**Published:** 2018-09-27

**Authors:** Gwang-Ju Jang, Mi Jeong Sung, Haeng Jeon Hur, Miyoung Yoo, Jung Hoon Choi, In Koo Hwang, Sanghee Lee

**Affiliations:** ^1^Korea Food Research Institute, Jeollabuk, Republic of Korea; ^2^Department of Anatomy, College of Veterinary Medicine, Kangwon National University, Gangwon, Republic of Korea; ^3^Department of Anatomy and Cell Biology, College of Veterinary Medicine, and Research Institute for Veterinary Science, Seoul National University, Seoul, Republic of Korea

## Abstract

Hyperlipidemia is a risk factor for atherosclerotic cardiovascular disease and is a major public health concern.* Allium hookeri* (AH) is an* Allium* species containing high levels of bioactive organosulfur compounds such as methiin and cycloalliin. AH exerts hypolipidemic effects in animals fed a high-fat diet. However, there exists little information on the mechanisms underlying these effects. To address this issue, we used a metabolomic approach based on ultra-performance liquid chromatography quadrupole time-of-flight mass spectrometry to identify factors mediating the lipid-lowering effects of AH. Principal component and partial least-squares discriminant analyses of serum metabolome profiles revealed 25 metabolites as potential biomarkers for the effects of AH on lipid levels. These compounds were predominantly phospholipids, including phosphatidylcholines (PCs), lysoPCs, and lysophosphatidylethanolamines. Glycerophospholipid metabolism was identified as a significantly enriched pathway. These results provide mechanistic insight into the antihyperlipidemic effects of AH and evidence for its efficacy as a therapeutic agent.

## 1. Introduction

Hyperlipidemia is a major public health problem and a risk factor for coronary heart disease [1, 2]. A clinical diagnosis of hyperlipidemia is made based on observation of increased levels of serum triglyceride (TG), total cholesterol (TC), and low-density lipoprotein cholesterol (LDL-C) in combination with a reduction in high-density lipoprotein cholesterol (HDL-C) [3, 4]. Hyperlipidemia is most often treated with lipid-lowering drugs such as statins and fibrates, though their widespread use is limited by side effects and poor tolerance in some patients [5]. However, the antioxidant activity of some natural products may effectively improve lipid metabolism and thus offer an alternate treatment for hyperlipidemia. For example, natural products such as oat and guar have been shown to decrease the levels of blood lipids, inhibit low-density lipoprotein oxidation, and improve abnormal lipid metabolism [2].

*Allium* (family Liliaceae) is a genus of flowering plants that includes* A. cepa* (onions),* A. sativum* (garlic), and* A. schoenoprasum* (chives), all of which have long histories of consumption as medicinal foods [6]. Sulfur compounds are the main bioactive constituents of* Allium* species [7].* A. hookeri* (AH) is a terrestrial perennial herb widely grown in India, Myanmar, and, more recently, in southern regions of South Korea, whose root is used for medicinal purposes [8]. It has been reported to have anti-inflammatory, anticancer, and hypolipidemic effects [9, 10] that have been mainly attributed to its organosulfur and phenolic compounds [11]. AH has especially high levels of the sulfur-containing compounds S-methylcysteine sulfoxide (methiin) and cycloalliin when compared to other* Allium* species such as garlic, onion, and leek [12]. Previous studies have indicated that cycloalliin and methiin have fibrinolytic, antihyperlipidemic, and serum triglycerol-lowering activities and contribute to decreased TC, LDL, and very-LDL-C (VLDL-C) in hyperlipidemic rats [13–16]. However, the effects and mechanisms of action of AH in hyperlipidemia have not been previously reported.

Metabolomics technology enables the identification of biomarkers in biofluids, tissues, and cells that can be used for disease diagnosis and toxicological and nutrition analyses [17–20]. Ultra-performance liquid chromatography- (UPLC-) mass spectrometry (MS) has been applied to metabolomics profiling and biomarker/mechanism discovery in disease models. UPLC-MS has been used to analyze metabolites in the serum, liver, or urine of obese mice in order to identify obesity-associated changes in lipid and energy metabolism [21, 22]. MS^E^ (MS^Elevated  Energy^) has two scanning functions for data collection. First, a low collision energy scan provides precursor ion information; next, a high collision energy scan provides mass fragment ion information [23, 24]. Metabolomic analysis using UPLC quadrupole time-of-flight- (Q-TOF-) MS can effectively identify metabolites with better resolution and higher sensitivity than is possible with MS^E^. In the present study, we employ both MS^E^ and ultra-performance liquid chromatography coupled with TOF-MS to identify potential biomarkers of hyperlipidemia in hamsters with HFD-induced hyperlipidemia and investigate the putative factors mediating the hypolipidemic effects of AH.

## 2. Materials and Methods

### 2.1. AH Sample Preparation, Animal Experiments, and Blood Collection

AH plants were harvested in 2013 and were purchased from a farmer in Cheongju Province, Korea. The roots were blanched for 7 min at 80°C to inactivate enzymes. The samples were then crushed using a DH 850 laboratory blender (Oscar, Seoul, Korea) and packed in vacuum film before being stored at -80°C until use.

Male Golden Syrian hamsters (8 weeks old) were purchased from Charles River Korea (Seoul, Korea) and housed at Kangwon National University at a constant temperature (22–26°C) under a 12:12-h light/dark cycle with free access to water and food. After acclimation for 1 week, the hamsters were randomly assigned to one of the following three groups: control (CON; fed a normal diet for 13 weeks [n = 6]), hyperlipidemic (HFD; fed an HFD for 13 weeks [n = 8]), and AH + hyperlipidemic (AH+HFD; fed an HFD for 13 weeks and treated with AH [n = 9]).

Composition of the HFD, by weight, was 20 % protein, 21 % fat, and 50 % carbohydrate. It was purchased from Research Diets Inc. (New Brunswick, NJ, USA). Hamsters in the AH+HFD group were orally administered AH dissolved in pure water at a dose of 0.2 g/kg/day once daily for 13 weeks. Animals in the CON and HFD groups were orally administered the same volume of water once daily for 13 weeks. The AH dose was chosen based on a similar study performed by Kim et al. in rats [25]. Blood samples were collected after being sacrificed and centrifuged at 3000 ×* g* for 10 min at 4°C and then stored at -80°C until analysis.

### 2.2. Serum Lipid Analysis

Serum TC, TG, LDL-C, and HDL-C levels were measured using an AU640 automatic analyzer (Olympus, Tokyo, Japan) according to the manufacturer's instructions.

### 2.3. UPLC-TOF/MS

A 200 *μ*L aliquot of serum was mixed with 600 *μ*L of cold acetonitrile (DAEJUNG, Gyeonggi-go, Korea) and vortexed. After centrifugation at 13000 rpm for 10 min, the supernatant was dried and the residue was reconstituted in 100 *μ*L of 20 % methanol (DAEJUNG, Gyeonggi-go, Korea). Samples were transferred to an injection vial prior to UPLC quadrupole TOF/MS analysis.

Chromatographic separation was performed on an Acquity BEH C18 UPLC column (1.7 *μ*m, 2.1 × 100 mm; Waters, Milford, MA, USA) at 40°C at a flow rate of 0.4 mL min^−1^. The mobile phase was composed of acetonitrile containing 0.1 % formic acid (Sigma-Aldrich, MO, USA) (A) and water containing 0.1 % formic acid (B) in a gradient of 2–100 % A over 24 min. The injection volume was 2 *μ*L.

A SYNAPY G2 high-definition mass spectrometer (Waters) was used with the electrospray ionization source operating in positive ion mode under the following conditions: capillary voltage, 2.5–3.0 kV; sample cone voltage, 30 V; and extraction cone voltage, 2.0 V. The nitrogen gas desolvation rate was set to 600 l/h at 350°C; the cone gas rate was 50 l/h; and the source temperature was 120°C.

Scan time and interscan delay were set to 0.15 and 0.02 s, respectively. Data were collected in centroid mode from m/z 100–1200. Data acquisition and processing were performed using MassLynx v.4.1 (Waters). Leucine-enkephalin (Waters, Manchester, USA) was used as the lock mass generating [M + H]+ (m/z 556.2771) to ensure accuracy during MS analysis. Dynamic range enhancement was applied throughout the experiment to ensure accurate mass measurement over a wide dynamic range.

### 2.4. Data Processing and Pattern Recognition Analysis

Raw data were analyzed using MassLynx v.4.1 software (Waters). The collected data included normalized peak intensity, exact mass, and retention time. The data matrix was then subjected to principal component analysis (PCA) and partial least-squares discriminant analysis (PLS-DA) for pattern identification using EZinfo v.2.0 software (Umetrics, Umeå, Sweden). To screen for biomarkers, differences between the groups were evaluated using Student's t-test and SPSS software (SPSS Inc., Chicago, IL, USA). P values less than 0.05 were considered significant. Potential biomarkers were assigned according to MS^E^ (Waters) fragment ion information and interpreted through comparisons to the Human Metabolome Database (http://www.hmdb.ca), LIPIDMAPS (http://www.lipidmaps.org) database, and METLIN (https://metlin.scripps.edu/) database. Metabolomics Pathway Analysis (MetPA) was also performed (http://www.metaboanalyst.ca/).

## 3. Results

### 3.1. Serum Lipid Analysis

Serum concentrations of TC, TG, and LDL-C were 5.3 ± 1.1, 3.3 ± 0.4, and 5.3 ± 1.1 mmol/L, respectively, in the CON group; these levels were significantly higher in the HFD group, at 7.7 ± 2.7 (P < 0.05), 6.8 ± 1.99 (P < 0.001), and 7.7 ± 2.7 (P < 0.05) mmol/L, respectively. HDL-C levels did not differ significantly between HFD (4.9 ± 0.3 mmol/L) and CON (4.6 ± 0.6 mmol/L) hamsters. AH treatment significantly reduced TC (5.5 ± 0.7 mmol/L, P < 0.05), TG (3.4 ± 0.2 mmol/L, P < 0.001), and LDL-C (5.5 ± 0.7 mmol/L, P < 0.05) levels similar to the CON group. Together, these data indicate that HFD-induced hyperlipidemia was successfully recapitulated in the HFD hamsters and that AH treatment effectively reversed this condition.

### 3.2. Multivariate Analysis of UPLC-QTOF-MS Data

**Figure 1:**
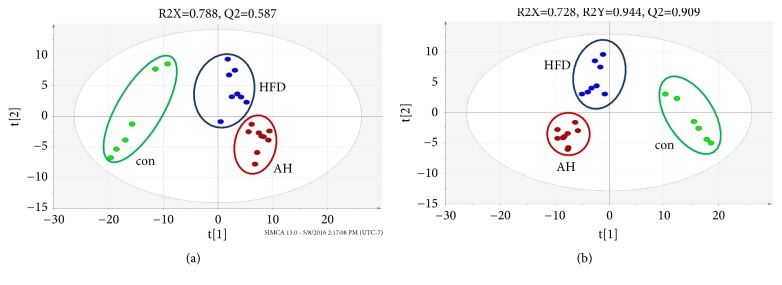
PCA score plot (a) and PLS-DA score plot (b) of serum samples analyzed by UPLC-Q-TOF-MS in positive mode. Normal control group (green), HFD group (blue), and HFD+AH (red).

MS data including retention time, peak intensity, and exact mass were imported to MassLynx software for statistical analyses. PCA and PLS-DA approaches are frequently used to identify differences between experimental groups and variations contributing to resulting classifications. The PCA score plots revealed that the three experimental groups differed significantly from each other in the positive mode (Figure 1(a)).

PLS-DA was used to identify differences in serum metabolic profiles among the CON, HFD, and HFD+AH groups as well as potential biomarkers. The PLS-DA score plots revealed similarities in metabolite patterns [R^2^X(cum) = 0.728, R^2^Y(cum) = 0.944, Q^2^ = 0.909 in positive mode] (Figure 1(b)). Serum metabolites whose levels differed significantly between groups were identified based on a projection value > 0.7 in PLS-DA models and a P value < 0.05. In total, 25 discriminant serum metabolites were identified and their relative levels were converted to fold-change values. Structure was predicted by comparing the MS fragments with known metabolites in various databases (Table 1).

### 3.3. Metabolic Pathways and Correlations among Metabolites

**Figure 2:**
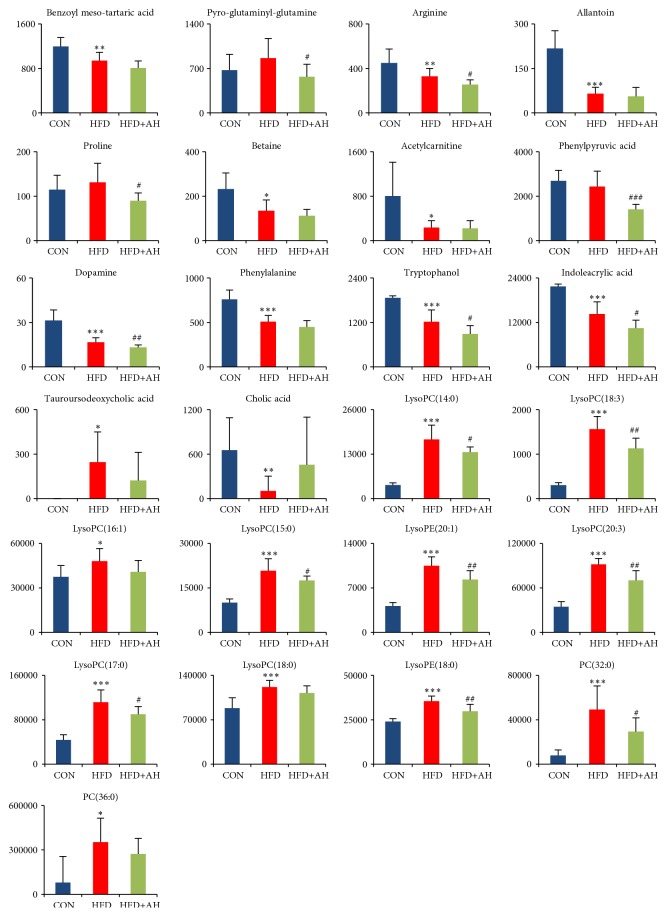
Comparison of different potential biomarkers among control group, HFD group, and HFD+AH group after administration. ^*∗*^p<0.05, ^*∗∗*^p<0.01, and ^*∗∗∗*^p<0.001 significant difference compared with control group; #p<0.05, ##p<0.01, and ###p<0.001 significant difference compared with HFD group. Y-axis: normalized relative intensity.

The 25 metabolites identified as potential biomarkers included amino acids (arginine, phenylalanine, proline, and pyro-glutaminyl-glutamine), organic acids (tartaric acid, phenylpyruvic acid, and indoleacrylic acid), bile acids (cholic acid and tauroursodeoxycholic acid), tryptophanol, betaine, dopamine, allantoin, acetylcarnitine, and glycerophospholipids (GPs) (Figure 2). Serum glutamine, proline, tauroursodeoxycholic acid, and lysoPCs levels were upregulated, whereas the other identified metabolites were downregulated, in the HFD group as compared to the CON group. The levels of 15 endogenous metabolites (pyro-glutaminyl-glutamine, arginine, proline, phenylpyruvic acid, dopamine, tryptophanol, indoleacrylic acid, lysoPC(14:0), lysoPC(18:3), lysoPC(15:0), lysophosphatidylethanolamine (lysoPE)(20:1), lysoPE(18:0), lysoPC(20:3), lysoPC(17:0), and PC(32:0)) in the serum samples were significantly affected by the HFD treatment and these perturbations could be partially reversed by AH treatment.

**Figure 3:**
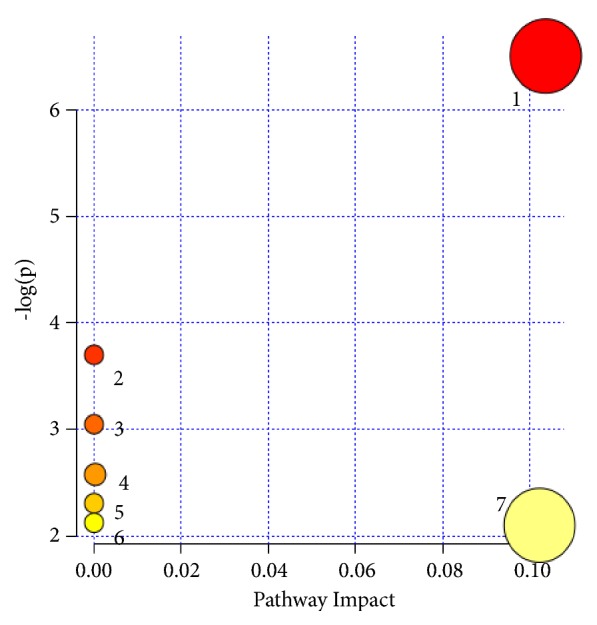
Summary of pathway analysis with MetPA. (1) Glycerophospholipid metabolism; (2) linoleic acid metabolism; (3) alpha-Linolenic acid metabolism; (4) primary bile acid biosynthesis; (5) arachidonic acid metabolism; (6) aminoacyl-tRNA biosynthesis; and (7) arginine and proline metabolism.

The MetPA identified seven pathways involved in the regulation of diet-induced hyperlipidemia, including glycerophospholipid metabolism, linoleic acid metabolism, alpha-linoleic acid metabolism, primary bile acid biosynthesis, arachidonic acid metabolism, aminoacyl-tRNA biosynthesis, and arginine and proline metabolism (Table 2). Alterations in these pathways in the HFD hamsters indicated that multiple interconnected metabolic pathways were likely involved in the alterations seen for some metabolites. The identified pathways were mainly related to glycerophospholipid metabolism, based on their impact (value > 0.1) and number of hits (value > 2) (Table 2, Figure 3).

## 4. Discussion

Hyperlipidemia is a disorder associated with obesity that is characterized by elevated levels of lipids in the blood. As such, changes in the levels of lipid-related metabolites in the blood have traditionally been used as indicators of hyperlipidemia [26, 27].

AH contains diverse phenols and phytosterols and a greater abundance of organosulfur compounds such as methiin and cycloalliin than other* Allium* species [12]. Methiin is known to have antidiabetic and hypolipidemic effects [14, 15] and reverses the increases in, cholesterol, TG, phospholipids, and alkyl and alkenyl sulfoxides and their breakdown products caused by a high-cholesterol diet [15]. Cycloalliin has been reported to reduce the concentration of serum triacylglycerol [16]. However, the effects of AH on the metabolome have not been previously reported. Here we combined a metabolomics approach with PCA and PLS-DA to identify metabolites and pathways associated with hyperlipidemia. We found that treatment with AH lowered various biochemical indices in the serum, providing evidence for its therapeutic efficacy in the treatment of HFD-induced hyperlipidemia.

The metabolite interaction network constructed through our analyses showed that AH exerts protective effects against HFD-induced hyperlipidemia by restoring biomarker levels to normal values. As with the studies performed by Kim et al. [21], the metabolic changes we saw, such as decreased betaine and carnitines and increased arginine and lysoPCs, were linked to abnormal mechanisms caused by HFD intake. Among the identified metabolites, 11 lipids were found to be associated with GP, linoleic acid, and alpha-linoleic acid metabolism, among other pathways. We determined that GP metabolism plays an important role in HFD-induced hyperlipidemia based on its impact (value > 0.1) and number of hits (value > 2). GPs are phospholipid derivatives found in cell membranes [28] that play important roles in atherosclerosis, inflammatory diseases, and disorders of lipid metabolism [29–31]. LysoPC, which is produced by PC hydrolysis, is associated with abnormal phosphorylcholine levels and may be a more accurate biomarker for specific metabolic phenotypes than total lipid concentration. To detect abnormalities in lysoPC levels in our hamster model of hyperlipidemia, we compared relative peak areas between the CON and HFD groups. Nine lysoPCs and PCs and two lysoPEs were upregulated in hamsters fed an HFD. The difference in lysoPC levels between the HFD and CON groups suggests that changes in lysoPC levels may underlie the pathogenesis of hyperlipidemia.

It was previously reported that methiin treatment abolished the increase in serum phospholipids induced by a high-cholesterol diet [15]. In the present study, the levels of five lysoPCs and PC [lysoPC(14:0), lysoPC(18:3), lysoPC(15:0), lysoPC(20:3), lysoPC(17:0), and PC(32:0)] and two lysoPEs [lysoPE(20:1) and lysoPE(18:0)] that were altered in HFD hamsters recovered to normal values upon AH administration.

*β*-Oxidation induced by an increase in saturated fatty acid levels in the liver and blood results in increased expression of high-mobility group-coenzyme A (HMG-CoA) reductase [32], which is required for the generation of energy in the tricarboxylic acid cycle or transformation of excess ketone bodies and cholesterol in the blood [33]. Downregulation of HMG-CoA attenuates cholesterol biosynthesis and concentration in the liver. Thus, AH may lower serum cholesterol by decreasing lysoPC levels, which in turn leads to a reduction in HMG-CoA reductase expression.

Bile acid is produced by the catabolism of cholesterol by cholesterol-7a-hydroxylase in the liver. The primary bile acids are cholic and chenodeoxycholic acid, while deoxycholic and lithocholic acid are secondary bile acids [34]. Cholic acid is known to prevent and reverse obesity, insulin resistance, and glucose tolerance. Cholic acid reduced the amount of white/brown adipose tissue and restored a normal body weight in obese mice on an HFD [35]. We found here that the cholic acid level was increased in the HFD+AH group, suggesting an alteration in bile acid production.

## 5. Conclusion

In this study, the serum lipid-regulating effects of AH in hamsters with HFD-induced hyperlipidemia was evaluated using a metabolomics approach. We found that TG, TC, and LDL-C levels were decreased in these hamsters relative to controls. We identified 25 metabolites whose levels were altered by an HFD and restored by administration of AH. These included lysoPCs related to glycerophospholipid, linoleic acid, and alpha-linoleic acid metabolism, among other pathways. Our results indicate that AH effectively reverses the increase in serum lipid levels caused by an HFD and can potentially inhibit the progression of hyperlipidemia to more severe conditions such as atherosclerotic cardiovascular disease.

## Figures and Tables

**Table 1:** UPLC-Q-TOF-MS analysis of identification results of potential biomarkers in serum measured by LC-QTOF/MS.

No.	RT^(a)^	Identified Ion (m/z)	VIP^(b)^	Adduct	Elemental composition	Metabolites	Hyperlipidemic group vs control group		AH-treated group vs hyperlipidemic group	
							FC^(c)^	p-value^(d)^	FC	p-valued
1	0.92	219.0271	0.238	[M+H-2H2O]+	C11H10O7	Benzoyl meso-tartaric acid	0.79	9.94×10-3	0.86	6.72×10-2
2	0.93	280.0922	0.331	[M+Na]+	C10H15N3O5	Pyro-glutaminyl-glutamine	1.28	2.47×10-1	0.66	3.33×10-2
3	0.94	175.1194	0.181	[M+H]+	C6H14N4O2	Arginine	0.73	4.12×10-2	0.78	1.81×10-2
4	0.98	203.0148	0.153	[N+2Na-H]+	C4H6N4O3	Allantoin	0.30	2.09×10-5	0.86	4.81×10-1
5	1.02	160.0350	0.118	[M+2Na-H]+	C5H9NO2	Proline	1.14	4.51×10-1	0.68	1.81×10-2
6	1.16	162.0505	0.121	[M+2Na-H]+	C5H11NO2	Betaine	0.58	1.07×10-2	0.83	2.52×10-1
7	1.63	204.1233	0.258	[M+H]+	C9H17NO4	Acetylcarnitine	0.29	2.94×10-2	0.94	8.19×10-1
8	2.14	165.0550	0.689	[M+H]+	C9H8O3	Phenylpyruvic acid	0.90	4.51×10-1	0.58	7.35×10-4
9	2.41	176.0672	0.052	[M+Na]+	C8H11NO2	Dopamine	0.53	1.88×10-4	0.79	9.45×10-3
10	3.01	166.0868	0.213	[M+H]+	C9H11NO2	Phenylalanine	0.67	1.65×10-4	0.88	1.02×10-1
11	3.85	144.0811	0.406	[M+H-H2O]+	C10H11NO	Tryptophanol	0.65	3.82×10-4	0.73	2.47×10-2
12	3.85	188.0714	1.400	[M+H]+	C11H9NO2	Indoleacrylic acid	0.66	1.54×10-4	0.73	1.17×10-2
13	9.54	464.2829	0.217	[M+H-2H2O]+	C26H45NO6S	Tauroursodeoxycholic acid	-	1.28×10-2	0.50	2.14×10-1
14	11.13	373.2740	0.355	[M+H]+	C24H40O5	Cholic acid	0.16	8.01×10-3	4.46	1.56×10-1
15	13.51	468.3094	1.539	[M+H]+	C22H46NO7P	LysoPC(14:0)	4.38	5.65×10-6	0.78	2.47×10-2
16	13.85	518.3246	0.790	[M+H]+	C26H48NO7P	LysoPC(18:3)	5.20	3.83×10-8	0.72	1.29×10-2
17	14.04	494.3251	1.142	[M+H]+	C24H48NO7P	LysoPC(16:1)	1.28	3.28×10-2	0.84	8.54×10-2
18	14.48	482.3246	1.325	[M+H]+	C23H48NO7P	LysoPC(15:0)	2.08	4.09×10-5	0.84	3.69×10-2
19	14.98	508.3406	1.089	[M+H]+	C26H54NO6P	LysoPE(20:1)	2.53	2.31×10-7	0.79	6.29×10-3
20	15.38	546.3565	3.406	[M+H]+	C28H52NO7P	LysoPC(20:3)	2.66	8.03×10-9	0.76	1.04×10-3
21	16.25	510.3562	2.007	[M+H]+	C25H52NO7P	LysoPC(17:0)	2.55	3.55×10-5	0.81	3.35×10-2
22	17.12	524.3714	1.906	[M+H]+	C26H52NO7P	LysoPC(18:0)	1.38	5.76×10-4	0.92	3.13×10-2
23	17.44	482.3245	1.728	[M+H]+	C23H48NO7P	LysoPE(18:0)	1.48	1.59×10-6	0.84	4.10×10-3
24	21.28	734.5687	3.244	[M+H]+	C40H80NO8P	PC(32:0)	6.24	6.03×10-4	0.60	2.43×10-1
25	21.73	834.6012	5.101	[M+H]+	C44H88NO8P	PC(36:0)	4.42	1.10×10-2	0.77	1.19×10-2

^(a)^Retention time.

^(b)^Variable importance in projection values was obtained from partial least square-discriminant analysis (PLS-DA) model.

^(c)^Relative levels of metabolites were converted into fold-changes.

^(d)^ *p* values were calculated from a one-way ANOVA.

**Table 2:** Result from ingenuity pathway analysis with MetPA.

Pathway	Total	hits	p	-log(p)	Holm p	FDR	impact
Glycerophospholipid metabolism	39	2	0.0015041	6.4996	0.12033	0.12033	0.104
Linoleic acid metabolism	15	1	0.024711	3.7005	1.0	0.98842	0.000
alpha-Linolenic acid metabolism	29	1	0.047358	3.05	1.0	1.0	0.000
Primary bile acid biosynthesis	47	1	0.075893	2.5784	1.0	1.0	0.000
Arachidonic acid metabolism	62	1	0.099179	2.3108	1.0	1.0	0.000
Aminoacyl-tRNA biosynthesis	75	1	0.119	2.1286	1.0	1.0	0.000
Arginine and proline metabolism	77	1	0.12202	2.1036	1.0	1.0	0.102

The total is number of compounds in the pathway; the hits are the actually matched number from the user uploaded data; the raw p is the original p value calculated from the enrichment analysis; the Holm p is the p value adjusted by Holm-Bonferroni method; and the impact is the pathway impact value calculated from pathway topology analysis.

## Data Availability

The data used to support the findings of this study are included within the article.
